# Exploring the Diagnostic Accuracy of the KidFit Screening Tool for Identifying Children with Health and Motor Performance-Related Fitness Impairments: A Feasibility Study

**DOI:** 10.3390/ijerph17030995

**Published:** 2020-02-05

**Authors:** Nikki Milne, Gary M Leong, Wayne Hing

**Affiliations:** 1 Faculty of Health Sciences and Medicine, Bond Institute of Health and Sport, Bond University, Gold Coast 4226, Australia; whing@bond.edu.au; 2 Department of Paediatrics, Nepean Blue Mountains Family Metabolic Health and Paediatric Diabetes Services, Nepean Hospital and the Nepean Charles Perkins Centre Research Hub, Kingswood 2747, Australia; celebrations1@mac.com

**Keywords:** KidFit, screening, health, fitness, motor proficiency, children, obesity

## Abstract

Child obesity is associated with poor health and reduced motor skills. This study aimed to assess the diagnostic accuracy of the KidFit Screening Tool for identifying children with overweight/obesity, reduced motor skills and reduced cardiorespiratory fitness. Fifty-seven children (mean age: 12.57 ± 1.82 years; male/female: 34/23) were analysed. The Speed and Agility Motor Screen (SAMS) and the Modified Shuttle Test-Paeds (MSTP) made up the KidFit Screening Tool. Motor Proficiency (BOT2) (Total and Gross) was also measured. BMI, peak-oxygen-uptake (VO2peak) were measured with a representative sub-sample (n = 25). Strong relationships existed between the independent variables included in the KidFit Screening Tool and; BMI (R^2^ = 0.779, *p* < 0.001); Gross Motor Proficiency (R^2^ = 0.612, *p* < 0.001) and VO2peak (mL/kg/min) (R^2^ = 0.754, *p* < 0.001). The KidFit Screening Tool has a correct classification rate of 0.84 for overweight/obesity, 0.77 for motor proficiency and 0.88 for cardiorespiratory fitness. The sensitivity and specificity of the KidFit Screening Tool for identifying children with overweight/obesity was 100% (SE = 0.00) and 78.95%, respectively (SE = 0.09), motor skills in the lowest quartile was 90% (SE = 0.095) and 74.47% (SE = 0.064), respectively, and poor cardiorespiratory fitness was 100% (SE = 0.00) and 82.35% (SE = 0.093), respectively. The KidFit Screening Tool has a strong relationship with health- and performance-related fitness, is accurate for identifying children with health- and performance-related fitness impairments and may assist in informing referral decisions for detailed clinical investigations.

## 1. Introduction

The rising prevalence of obesity over recent decades and the current rates of child obesity in Australia and internationally remain a concern for health and education workers and policy makers alike. Child overweight and obesity is associated with poor health outcomes such as insulin resistance, type 2 diabetes mellitus (T2DM), hypertension, dyslipidaemia and fatty liver disease [1]. Further association has been shown with idiopathic intracranial hypertension [2] and obstructive sleep apnoea [3,4,5], which can result in daytime somnolence and neurocognitive deficits such as reduced concentration and poor memory [6,7]. Some researchers believe that these neurocognitive deficits may be responsible for the relationship between Body Mass Index (BMI), an indicator of excess adiposity among children [8], and academic achievement [9]; however, recent research indicates an absence of a relationship between school literacy and numeracy and percentage body fat, after adjusting for socioeconomic and cultural status [10]. Conversely, physical activity and cardiorespiratory fitness (CRF) have both been positively linked to cognitive processes and brain function in children, with aerobically fitter children performing better on cognitive function tasks [11,12].

Physical activity has been firmly associated with health-related fitness [13], as well as motor proficiency [14,15,16]. However, to be physically active, children require the underlying motor skills that enable them to take up physical activity in order to develop or maintain good health-related fitness and consequently enhance opportunities for positive educational outcomes.

With exercise capacity being known to decrease all-cause mortality [17], independent of adiposity [18], a screening tool that does not require physical body measurements such as BMI, but rather focuses on CRF and motor performance-related fitness could be used by health and education professionals to identify children who could benefit from specialist assessment and interventions relating to motor proficiency and physical fitness.

The KidFit Screening Tool has been carefully designed for this purpose and includes the combination of two simple measures: (i) Modified Shuttle Test-Paeds (MSTP)—a health-related CRF measure [19]—and (ii) the Speed and Agility Motor Screen (SAMS) [20], with both components being known to individually challenge overweight and obese children [21]. Therefore, in order to test the feasibility of using the KidFit Screening Tool to identify children who could benefit from specialised assessment and interventions relating to motor proficiency and physical fitness, the aims of this study were to firstly explore the relationship between the KidFit Screening Tool and reference measures of health and performance-related fitness in children and, secondly, to investigate the diagnostic accuracy (including sensitivity and specificity) of the KidFit Screening Tool for identifying children with overweight or obesity, reduced motor skills and reduced CRF.

## 2. Materials and Methods

Data from fifty-seven children (age: 6–17 years; male/female: 34/23) who were recruited via community flyers, advertisement to local schools and word of mouth to participate in two previously reported studies [19,20] were utilised for analysis in the present study. To be eligible for inclusion to the present study, children needed to be aged between 5 and 17 years and attending school at the time of testing. Children who were unable to complete the testing due to mobility-limiting orthopaedic or neurological conditions were excluded from the present study. Informed participant assent and parent/legal guardian consent were obtained for all children who participated. The Bond University Human Research Ethics Committee reviewed and approved the experimental protocols from which the data in this study were collected (RO1601, RO1019). The first study specifically recruited children who were overweight or obese as well as a healthy weight reference group, whereas the second study recruited children of “all shapes, sizes and fitness levels”. All persons involved with collecting data in these studies were trained by the primary author and assessed for the reliability of the testing methods prior to data collection. All research was conducted in accordance with the Declaration of Helsinki (1964). The STARD Statement for reporting studies of diagnostic accuracy [22] was used to guide the methods and reporting in this study.

### 2.1. Experimental Design

All measures were undertaken at either the participant’s school or Bond University research lab. Prior to their testing session, the participants were familiarised with the experimental protocol and the equipment to be used. Parents were invited to observe their child’s participation. Children who met the above stated inclusion and exclusion criteria and had completed the measures of Motor Proficiency (BOT2) (Total and Gross), Speed and Agility Motor Screen (SAMS) and the Modified Shuttle Test-Paeds (MSTP) were included in this study (n = 57). The measures of height, weight and peak oxygen uptake (VO2peak) were also undertaken with participants who attended the appointments at Bond University research labs (n = 25), as these measures were not approved to be taken in school environments. Total Motor Proficiency and Gross Motor Proficiency were operationally measured by the Bruininks Oseretsky Test of Motor Proficiency, 2nd Edition (BOT2), which was used as the motor skills reference measure in this study. BMI and VO2 peak were the reference measures for health-related fitness and were measured in a subset of participants as the standard of classification for overweight/obesity and cardiorespiratory fitness respectively.

### 2.2. Procedures and Measures

#### 2.2.1. Standardised Motor Proficiency Measure (BOT2) (Reference Measure)

The Bruininks Oseretsky Test of Motor Proficiency, 2nd Edition (BOT2) is an internationally recognised discriminative normative reference tool that has been previously validated and found to be reliable for assessing the motor proficiency of children aged 4.5–17 years [23]. The BOT2 was used to assess the gross and overall (combined fine and gross) motor proficiency of children in this study to derive a Total Motor Proficiency score. Standardised instructions and scoring methods were applied for all BOT2 subtests as per the assessment manual [23] with the only exception being the order of testing, which was planned around the CRF measures. The BOT2 Total Motor Proficiency score was derived from all subtest raw scores including fine motor subtests (Fine Motor Precision, Fine Motor Integration, Manual Dexterity) and gross motor subtests (Upper Limb Coordination, Bilateral Coordination, Balance, Running Speed and Agility, Strength). The BOT2 Gross Motor score used for analysis in this study included the combined gross motor subtest raw scores of Bilateral Coordination, Balance, Running Speed and Agility and Strength. All BOT2 motor subtest scores were combined to determine a BOT2 percentile rank, and this information was used to sub-classify participants into BOT2 quartile groups ((1st Motor Quartile Group—0% to ≤25% (lowest); 2nd Motor Quartile Group—>25% to ≤50%; 3rd Motor Quartile Group—>50% to ≤75% and 4th Motor Quartile Group—>75% to ≤100% (highest)).

#### 2.2.2. Motor Skills Screening Tool (SAMS)

The Speed and Agility Motor Screen (SAMS) is a valid and reliable measure that has been previously reported to predict poor gross motor proficiency in overweight/obese children [20]. The SAMS is a quick-to-apply timed motor performance-related fitness measure which includes components of speed and agility, whole body coordination, core strength and balance. To complete the SAMS, the participants were instructed to move from standing with hands by their sides, to laying prone on a 5 cm thick mat with hands above their head and feet together, log roll 360 degrees, stand up and perform a jumping jack (2-stage start jump) and to do this as quickly as they could. All measures were taken by a qualified physiotherapist, a physiotherapy student under supervision or a physical education teacher. All participants were given a practice trial before a timed trial using a “ready, go” trigger. After 30 s rest, the participants were offered a second timed trial, and this was the SAMS time used for the present study.

#### 2.2.3. Anthropometric Measurements

Standing height was measured by a qualified physiotherapist (or physiotherapy student under supervision) at the top of tidal inspiration on a solid surface with children in bare feet, using a tape measure to the nearest 0.5 cm. Body mass was measured in kilograms using calibrated scales with the children wearing light clothing (e.g., shorts and shirt). The children were blinded to their weight measurements using a shield over the display. Height and weight measures were subsequently used to calculate BMI (raw scores and percentiles) using CDC growth charts [24].

#### 2.2.4. Gold Standard Cardiorespiratory Fitness (CRF) Measure (VO2peak)

Peak oxygen uptake (VO2peak) was determined on a motor-driven treadmill (‘Valiant’; Lode B.V., Groningen, The Netherlands) via an incremental exercise test to volitional fatigue. The protocol involved an accredited exercise physiologist predetermining a preferred walking speed of between 4.0 and 5.0 km/h at 0% grade to start the test and then increasing the speed every 60 s until the participant reached their previously determined comfortable running speed, which was between 6.0 and 8.0 km/h for all participants. The treadmill grade was then increased every 60 s by 1% for younger children (≤12 years) and 2% for adolescent (>13 years) participants until the participant could no longer continue. Breath-by-breath values for oxygen uptake (VO2), minute expired ventilation and carbon dioxide output (VCO2) were measured using a calibrated open-circuit metabolic measurement system (Ultima CPX, Medical Graphics Corporation, St Paul, MN, USA). A 12-lead ECG (Cardio Perfect, Welch Allyn Inc., Skaneateles Falls, NY, USA) was used to monitor cardiac rhythm throughout the test. To calculate the peak exercise values, the average of the two highest 30 s values measured before test discontinuation were used.

#### 2.2.5. Cardiorespiratory Fitness Screening Tool (MSTP)

The Modified Shuttle Test-Paeds (MSTP) is a valid field measure of CRF with a high predictive validity for estimating VO2peak in children [19]. The MSTP involved the children running a straight 10 m shuttle, picking up 1 hand-held bean bag from a tray on the ground, turning around and returning to the start point to place the bean bag into an identical size tray on the start line, and repeating this as many times as they could in 3 min. Strong verbal encouragement was offered to the children in the test to enhance the likelihood of maximal effort being achieved. For every bean bag returned to the tray, the child received one (1) point and for any additional bean bag that the child may have picked up but not yet returned to the tray they received a further half (1/2) point. A registered physiotherapist or physical education teaching completed the MSTP measures after training to ensure reliability.

#### 2.2.6. KidFit Screening Tool

The KidFit Screening Tool was made up of two of the above mentioned measures: (i) SAMS, using a previously reported cut-off time of ≥5.43 s to indicate concerns with motor skills, in particular speed and agility [20], and (ii) MSTP, using a previously determined equation for predicting VO2max (mL/kg/min) in children [19] which then allowed the CRF of children to be classified as very poor, poor, fair, good, excellent or superior (cut-off values for those considered to have reduced CRF ≤ 34.9 mL/kg/min for girls and ≤45.1 mL/kg/min for boys) [25]. Children with a MSTP predicted VO2max in the very poor, poor, or only fair categories were indicated as needing further assessment to investigate reasons for reduced CRF. When the KidFit Screening Tool was applied, any child who took ≥5.43 s to complete the SAMS and/or had a MSTP predicted VO2max under the above mentioned cut-off values would be identified as KidFit positive (+ve)—needing further detailed assessment by a suitably qualified health professional (e.g., paediatric physiotherapist) to work towards improving a child’s health and performance-related fitness to prevent the onset of associated chronic disease. If a child completes the SAMS in less than 5.43 s and had an MSTP predicted VO2max over the above-mentioned thresholds, then they would be identified as KidFit negative (−ve)—not requiring further investigations related to health- or performance-related fitness.

#### 2.2.7. Statistical Analysis

Means and standard deviations were calculated for all measures collected. Percentages and frequencies were also calculated for all physiological and anthropometric measures. Independent samples t tests were used to determine if differences existed between girls and boys for each of the health- and performance-related fitness measures. Multiple linear regression analysis, applying a simultaneous enter method, was used to determine if a relationship existed between the independent variables that make up the KidFit Screening Tool (SAMS and MSTP) and BMI, Motor Proficiency (Total and Gross) and VO2peak, and this was repeated with age and gender included in the model, to determine the concurrent validity of the KidFit Screening Tool. If the KidFit Screening Tool was significantly associated with the health- and performance-related fitness variables, then the diagnostic accuracy of the KidFit Screening Tool was explored to investigate its discriminative power to accurately identify children with BMI above the 85th percentile, Motor Proficiency in the first quartile and reduced CRF. To assess the diagnostic accuracy of the KidFit Screening Tool, the sensitivity, specificity, positive and negative predictive values, positive likelihood ratios (LR+), negative likelihood ratios (LR−), efficiency rates (ER) and odds ratios (OR) were calculated for the combined tests that make up the KidFit Screening Tool, applying methods previously utilised [26]. Receiver operating characteristic (ROC) curves were plotted for each of the state variables (BMI, Total Motor Proficiency and VO2peak) so that the balance of sensitivity and specificity could be assessed using the area under the curve (AUC), applying methods published in previous research [27]. These measures assisted in determining the diagnostic accuracy of the KidFit Screening Tool for identifying children with BMI percentiles in the overweight or obese category, Motor Proficiency in the first (lowest) quartile and reduced cardiorespiratory fitness (i.e., VO2peak in the very poor, poor or fair categories, using cut-off values provided by The Cooper Institute for Aerobics Research [25]. The narrative analysis for diagnostic decision making was also applied using the criteria reported previously by Thorne and colleagues [28], as outlined in the Table 1.

Fisher’s exact test was performed to determine if the classification variables using cut-off thresholds determined in previous studies [19,20] ((BMI percentiles in the overweight or obese categories, Total Motor Proficiency in the first (lowest) BOT2 quartile and reduced CRF (VO2peak in the fair, poor or very poor which equates to the cut-off values of ≤34.9 mL/kg/min for girls and ≤45.1 mL/kg/min for boys)) were significantly associated with the KidFit Screening Tool classification. Statistics were analysed with SPSS for Windows (Version 21.0, IBM Corp, Armonk, NY, USA) in addition to a spreadsheet for the calculation of comprehensive statistics for the assessment of diagnostic tests and inter-rater agreement [29]. Significance level was set at *p* < 0.05.

## 3. Results

Fifty-seven children (Mean age: 12.57 ± 1.82 years) participated in this study with a mean total motor percentile rank in the average range, but 10 (17.54%) children were in the lowest motor quartile (0 to ≤25%). BMI was calculated for just under half (n = 25) of children who participated in this study and of these children the majority (n = 16, 64%) were in the healthy weight range, with 12% (n = 3) underweight and 24% (n = 6) overweight or obese. This figure is consistent with the Australian reference data of approximately one in every four children being overweight or obese [30].

The same 25 children whose BMI was measured also undertook an incremental exercise test to determine their peak oxygen uptake (VO2peak). One child, however, was unable to complete the treadmill test due to a cardiac anomaly being detected on 12-lead ECG early in the test administration and this measure was removed from analyses. Other than this, all data from the 57 participants were included for analysis. The mean VO2peak for girls (41.32 ± 3.55 mL/kg/min) is considered to be in the ‘excellent’ range, whereas the mean VO2peak for boys (45.06 ± 12.53 mL/kg/min) was considered to be in the ‘good’ range, using reference data from The Cooper Institute for Aerobics Research [25].

There were no significant differences between boys and girls in this study for any raw scores in the health- or performance-related measures. However, there was a significant difference in age between girls and boys (t = −2.59, DF = 55, *p* = 0.012) with the mean age of girls being 1.21 years younger than boys in the study. Additionally, there was a significant difference between girls and boys in the Total Motor Proficiency Percentile Ranks (t = 2.42, DF = 55, *p* = 0.019), where female participants had a mean Total Motor Proficiency percentile rank 19 points higher than the male participants after age was factored into the calculation, indicating that overall the girls in this study had significantly better motor skills for their age than the boys.

Table 2 provides the means and standard deviations for the health- and performance-related fitness characteristics of participants included in this study. The relationship between the KidFit Screening Tool (i.e., MSTP and SAMS) and BMI, Motor Proficiency (Total and Gross), and VO2peak with and without the age and gender of children included in the model is provided in Table 3.

Table 4 details the diagnostic accuracy of the KidFit Screening Tool for identifying children with overweight and/or obesity, Total Motor Proficiency in the 1st (Lowest) Motor Quartile according to BOT2 results and low CRF (VO2peak in the very poor, poor or fair categories: Girls cut-off = MSTP Predicted VO2peak ≤ 34.9 mL/kg/min; Boys cut-off = MSTP Predicted VO2peak ≤ 45.1 mL/kg/min).

Table 5 provides the output from the Receiver Operating Curves (ROC), including the area under the curve (AUC); a global measure of diagnostic accuracy. The closer the score is to 1.0, the better the discriminative power of a tool. The results in Table 5 show that that KidFit Screening Tool has a ‘moderate to high’ accuracy for identifying children with overweight or obesity, motor skills in the first (lowest) Motor Quartile and/or poor CRF.

## 4. Discussion

The present study explored the feasibility of using a simple screening tool (i.e., The KidFit Screening Tool) to identify children with reduced health- and performance-related fitness. In doing so, this study initially aimed to determine if a relationship existed between the collective components of the newly designed KidFit Screening Tool (i.e., MSTP and SAMS) and the dependent health and performance-related fitness variables of BMI, CRF and Motor Proficiency (Total and Gross) in children. Once these relationships were confirmed, the present study aimed to assess the diagnostic accuracy (including sensitivity and specificity) of the KidFit Screening Tool to identify children who are overweight or obese, have reduced motor skills and/or have reduced CRF.

The results of this study indicate that the KidFit Screening Tool has a strong and significant relationship with the health-related fitness measures of BMI and VO2peak. Additionally, the KidFit Screening Tool was found to have a strong and significant relationship with Gross Motor Proficiency, but only a moderately strong relationship with Total Motor Proficiency (which is inclusive of gross and fine motor skills). These results address the first of our study aims, suggesting that the combined measures in the KidFit Screening Tool are related to health- and performance-related fitness in children. These results are important as they suggest that the measures included in the KidFit Screening Tool (MSTP and SAMS), when used together, are valid indicators of health- and performance-related fitness, and are feasible for using in the KidFit Screening Tool for the purpose as suggested above. Additionally, despite the KidFit only being a screening tool, our findings were consistent with those of a recent systematic review with meta-analysis, which demonstrated a clear relationship between motor competence and physical fitness in children of varying ages, leading the authors to conclude that motor competence and physical fitness (including cardiorespiratory fitness) are important factors for promoting positive trajectories of health over time [31]. These statements further support the screening of these measures and offering intervention when required to ensure good health over time.

With regards to the second of our study aims, the results of this research study provide preliminary evidence that the KidFit is a feasible tool for screening children, which is moderately to highly accurate in identifying those who are overweight or obese who have motor skills in the lowest quartile and/or poor cardiorespiratory fitness. With sensitivity ranging from 90% to 100% and specificity ranging from 74% to 82%, in addition to a correct classification rate of 0.77–0.88 for a test that takes less than 5 min to complete, the KidFit Screening Tool may be as good, and possibly more feasible than other previously reported screening measures for identifying children with poor health and motor performance-related fitness. For example, Kroes and colleagues [32] reported on Maastricht’s Motor Test (MMT), a screening test for distinguishing children with abnormal motor behaviour from those with normal motor behaviour, reporting 86% sensitivity and 70% specificity for the MMT. Although there are many standardised motor assessments for children with the concurrent and predictive validity and reliability documented, there is little documented research investigating the sensitivity and specificity of the measures for accurately identifying children and adolescents with poor motor skills. Conversely, a number of CRF measures and classifications have been developed to assist with identifying children with higher cardiovascular disease (CVD) risk factors, with sensitivity documented between 33.3% and 100% and specificity rates between 34.0% and 92.1% [33]. The KidFit Screening Tool is at the upper end of these values for both sensitivity and specificity, adding weight to the worth and feasibility of the tool for screening purposes. Furthermore, the measures which make up the KidFit Screening tool are quick to administer and score, do not require expensive equipment and do not require extensive training or qualifications to utilise with children. In comparison, to complete a standardised motor assessment and/or VO2max testing with children (which may be appropriate for later assessments), expensive equipment is required, children need to be assessed in a standardised testing environment, the examiners need extensive training and it takes well over an hour to complete the testing and score the results for each child.

Impaired motor skills that limit a child’s ability to be physically active can reduce CRF [34] and CRF is associated with poor health outcomes, leading to chronic disease [1]. Additionally, aerobically fitter children are more likely to achieve better results in cognitive function tasks [11,12], have more efficient neurocognitive processing, and larger hippocampal and basal ganglia volumes, compared to children with lower cardiorespiratory fitness [35] and children with a healthy weight tend to attain better academic outcomes at school [9]. For these reasons, the early identification of children with poor health and motor performance-related fitness is critical. Early identification can assist with timely referral to specialised services for intervention, to prevent or ameliorate the onset of chronic disease and/or the associated adverse psychosocial/learning outcomes for the child. The KidFit Screening Tool can be utilised with children individually or in large groups and may be considered a feasible tool to assist with this early identification. It is a quick- and easy-to-administer test with high sensitivity and specificity, supporting the suitability of the KidFit Screening Tool to be used by persons who have access to observing children during physical activity, such as Physical Education (PE) teachers, coaches or health clinicians, to assist with screening the health and motor performance-related fitness of children when the context is appropriate to do so. The implementation of such screening could help with making decisions regarding early referrals to specialised services (e.g., paediatric physiotherapists) for (i) the detailed investigation of underlying reasons for reduced motor proficiency or CRF and (ii) intervention to prevent the onset of associated chronic disease, or any negative psychosocial/learning outcomes.

The present study has some notable limitations that should be considered when interpreting the results. Firstly, the study cohort is small relative to the age span investigated, with fewer female participants than males and females having higher Total Motor Proficiency (once adjusted for age) than male participants, which may limit the generalisability of the study findings to wider populations. To counteract this limitation, we controlled for age and gender in the multiple regression analyses. This small sample may further impact the results, with some children in the study being morbidly obese, possibly creating spectrum bias, impacting the measures of test accuracy [22], and this should be considered when interpreting the results. Further to this limitation, due to this study exploring the feasibility and having no prior KidFit data, we did not complete a power analysis for diagnostic accuracy tests. It is anticipated, however, that the data from this study will be useful for power analyses in future, larger cohort studies with the KidFit Screening Tool. Secondly, despite numerous studies reporting children’s CRF levels, including directly measured and predicted VO2max, there remains a lack of consensus amongst researchers and clinicians on the appropriate cut-off values for accurately identifying CVD risk factors in children, particularly very young children, and this limited our ability to access appropriate comparison data. For example, the VO2peak cut-off values for the KidFit Screening Tool in the present study (Girls: MSTP Predicted VO2peak ≤ 34.9 mL/kg/min; Boys: MSTP Predicted VO2peak ≤ 45.1 mL/kg/min) were derived from reference data produced by the Cooper Institute for Aerobics Research [25], which is based on children aged 13–19 years. These VO2max categories and cut-off values for identifying children with reduced CRF in this study are, however, consistent with the cut-off values identified by Bergmann and colleagues [33] in a review of procedures for the creation of CRF cut-off points for children and adolescents. Cut off values to accurately identify children and adolescents with an increased likelihood of developing CVD risk factors were shown to range between 33.0 and 40.1 mL/kg/min for females and 37.6 and 46.0 mL/kg/min for males [33], which are inclusive of the cut-off values used in the present study. Based on the above limitations, the authors caution users in interpreting the results of the KidFit Screening Tool as a standalone assessment and recommend that the results of the KidFit Screening Tool be interpreted as the first line of screening assessment only and should be considered in the context of further examination. Future research is warranted to explore the use of the KidFit Screening Tool with larger cohorts, using more refined age groups and according to sex.

## 5. Conclusions

The KidFit Screening Tool has a strong and significant relationship with health- and performance-related fitness measures of BMI, VO2peak and Gross Motor Proficiency after controlling for age and gender and is moderately to highly accurate for identifying children with and without overweight or obesity, motor skills in the lowest quartile and/or poor cardiorespiratory fitness. The information derived from the KidFit Screening Tool may be used to inform decisions regarding referral to specialised services for detailed investigation of underlying reasons for poor health- and/or performance-related fitness. The KidFit Screening Tool therefore is presented as a feasible and accurate screening tool which provides an important contribution to the early identification of poor health- and motor performance-related fitness in children so that users can instigate early intervention and prevention of associated chronic disease.

## Figures and Tables

**Table 1:** Criteria applied for levels of evidence for a reasonable diagnostic screening measure using the Area Under the Curve (AUC).

Level of Evidence	Criteria
Random Accuracy	AUC < 0.5
Poorly Accurate	AUC = 0.5–0.7
Moderately Accurate	AUC = 0.7–0.9
Highly Accurate	AUC = 0.9–1.0

AUC: Area under the curve.

**Table 2:** Means and standard deviations for health- and performance-related fitness measures.

Variable	Participant Numbers	Mean ± SD
MSTP (No.)	57	21.83 ± 2.93
MSTP Predicted VO_2_peak (mL/kg/min)	57	43.63 ± 9.16
VO2peak (mL/kg/min)	24	44.12 ± 11.02
VO2peak (mL/min)	24	2294.85 ± 828.85
Total Motor Percentile Rank (BOT2)	57	61.42 ± 30.46
SAMS (s)	57	4.68 ± 1.40
BMI raw score (kg/m^2^)	25	20.96 ± 8.75
BMI percentile	25	51.84 ± 33.94
BMI Z score	25	0.11 ± 1.40

Data presented as mean ± SD. MSTP: Modified Shuttle Test—Paeds, MSTP Predicted VO2max using the predictive equation provided in [19], VO2peak: peak oxygen uptake, BOT2: Bruininks Oseretsky Test of Motor Proficiency, 2nd Edition, SAMS: Speed and Agility Motor Screen, BMI: Body Mass Index. NOTE: Mean BMI raw scores and percentiles in addition to the VO2peak (mL/kg/min) have been previously published [19].

**Table 3:** Relationship between the combined variables in the KidFit Screening Tool (MSTP and SAMS) and BMI, VO2peak and Motor Proficiency (Total and Gross).

Model	Independent Variables	Dependent Variables			
		BMI (kg/m^2^)	BOT2 Total Motor (Raw Score)	BOT2 Total Gross Motor (Raw Score)	VO_2_peak (mL/kg/min)
		Coefficients of Determination (R^2^) (*p* value)			
Model 1 KidFit (MSTP and SAMS)		0.770 (<0.001)	0.494 (<0.001)	0.612 (<0.001)	0.754 (<0.001)
		Standardised Beta Coefficients—Model 1 (*p*-value)			
	SAMS	0.681	−0.106	−0.221	−0.101
		(<0.001)	(0.366)	(0.035)	(0.552)
	MSTP	−0.245	0.638	0.637	0.789
		(0.130)	(<0.001)	(<0.001)	(<0.001)
Model 2 KidFit (MSTP, SAMS, Age and Gender)		0.871 (<0.001)	0.543 (<0.001)	0.644 (<0.001)	0.813 (<0.001)
		Standardised Beta Coefficients—0Model 2 (*p*-value)			
	SAMS	0.595	−0.078	−0.201	−0.186
		(<0.000)	(0.509)	(0.057)	(0.277)
	MSTP	−0.321	0.674	0.663	0.719
		(0.026)	(<0.000)	(<0.001)	(<0.001)
	Age	0.333	0.169	0.140	−0.224
		(0.002)	(0.097)	(0.118)	(0.067)
	Gender	−0.056	−0.214	−0.165	0.273
		(0.579)	(0.042)	(0.075)	(0.038)

BMI: Body Mass Index. BOT2 Total Motor Proficiency: includes all subtest raw scores including Fine Motor Precision, Fine Motor Integration, Manual Dexterity, Upper Limb Coordination, Bilateral Coordination, Balance, Running Speed and Agility, Strength. BOT2 Total Gross Motor Proficiency: includes gross motor subtest raw scores including Bilateral Coordination, Balance, Running Speed and Agility, Strength. VO2peak: Directly measured peak oxygen consumption during a graded exercise test on a treadmill. Coefficients of determination are reported for models and standardised beta coefficients are reported as individual contributors to the model. Significance is set at *p* = 0.05.

**Table 4:** Diagnostic accuracy parameters of the KidFit Screening Tool for identifying health or performance-related fitness concerns in children.

Health or Performance-Related Fitness Measures	Cell Counts				Diagnostic Accuracy Parameters								
	TP	FN	FP	TN	Sensitivity (SE)	Specificity (SE)	PPV (SE)	NPV (SE)	LR+ (95% CI)	LR− (95% CI)	OR (95% CI)	ER	Fisher’s Exact Test *p* Value
BMI (≥85th Percentile) Overweight or Obese) (n = 25)	6	0	4	15	100% (0.00)	78.95% (0.093)	60% (0.153)	100% (0.00)	4.75 (1.99–11.35)	0.00 (-)	44.78 (2.10–956.84)	0.84	<0.001
Total Motor Proficiency (1st Motor Quartile) (n = 57)	9	1	12	35	90% (0.095)	74.47% (0.064)	43% (0.108)	97% (0.027)	3.53 (2.07–5.99)	0.13 (0.02–0.87)	26.25 (3.00 –229.34)	0.77	<0.001
Poor VO_2_peak (mL/kg/min) (n = 24)	7	0	3	14	100% (0.00)	82.35% (0.093)	70% (0.145)	100% (0.00)	5.67 (2.03–15.82)	0.00 (-)	62.14 (2.82–1367.82)	0.88	<0.001

Abbreviations: TP: true positives; FN: false negatives; FP: false positives; TN: true negatives; CI: confidence interval; PPV: positive predictive value; NPV: negative predictive value; LR+: positive likelihood ratio; LR−: negative likelihood ratio; OR: odds ratio; ER: Efficiency Rate. All *p*-values for the OR were <0.05 (significant).

**Table 5:** Output from the Receiver Operating Curves (ROC) for determining the accuracy of the KidFit Screening Tool for identifying children with overweight or obesity, motor skills in the lowest quartile and/or poor cardiorespiratory fitness.

State Variable	AUC	SE	CI	*p* Value
BMI (Percentile) Overweight/Obesity	0.895 **	0.063	0.771, 1.00	0.004
Total Motor Proficiency (BOT2 Percentile) (1st Quartile)	0.822 **	0.069	0.688, 0.957	0.001
Poor CRF (VO2peak)	0.912 **	0.060	0.794, 1.00	0.002

AUC: Area Under the Curve; SE: Standard Error; CI: Confidence Intervals; *p* values: Significance levels; VO2peak: Peak Oxygen Uptake as a measure of cardiorespiratory fitness (CRF) measured in mL/kg/min); BMI: Body Mass Index; ** *p* values are significant at the 0.01 level.
